# Severe Left Ventricular Outflow Tract Obstruction Following Arteriovenous Fistula Reopening in a Dialysis Patient With Left Ventricular Hypertrophy

**DOI:** 10.7759/cureus.81284

**Published:** 2025-03-27

**Authors:** Abbas Rachid, Batoul Chaaban, Malek Mohammed, Nina M Khalil, Hasan Kazma

**Affiliations:** 1 Internal Medicine, Lebanese University, Beirut, LBN; 2 Cardiology, Lebanese University, Beirut, LBN; 3 Cardiovascular Disease, Bahman University Hospital, Beirut, LBN; 4 Nephrology, Bahman University Hospital, Beirut, LBN

**Keywords:** arteriovenous fistula, end stage renal disease (esrd), high-output heart failure, left ventricular hypertrophy (lvh), left ventricular outflow obstruction (lvot), systolic anterior motion of mitral valve

## Abstract

Arteriovenous fistulas (AVFs) are the preferred vascular access for hemodialysis due to their durability and lower risk of complications than alternative access methods. However, AVFs can significantly impact cardiac function, particularly in patients with preexisting cardiovascular conditions. We present a case of a 56-year-old female with a history of hypertension and end-stage renal disease who developed recurrent hypotension, dizziness, and dyspnea following AVF reopening. The echocardiographic evaluation revealed severe left ventricular hypertrophy, systolic anterior motion of the mitral valve, significant mitral regurgitation, and left ventricular outflow tract (LVOT) obstruction with a high-pressure gradient. Despite medical management with beta-blockers, symptoms persisted, leading to the decision to close the AVF surgically. Following AVF closure, echocardiography showed a marked improvement in mitral regurgitation and resolution of LVOT obstruction, with the patient tolerating subsequent dialysis without complications. This case highlights the potential hemodynamic consequences of AVF reopening in patients with underlying cardiac pathology and emphasizes the need for careful cardiovascular evaluation.

## Introduction

Arteriovenous fistulas (AVFs) are widely regarded as the gold standard for vascular access in chronic hemodialysis due to their superior longevity, lower risk of infection, and reduced mortality compared to arteriovenous grafts (AVGs) and central venous catheters. However, AVFs can exert a substantial hemodynamic burden on the cardiovascular system despite these advantages. The creation of an AVF leads to a diversion of blood from the high-resistance arterial circulation into the low-resistance venous system, resulting in increased venous return, elevated cardiac output, and reduced systemic vascular resistance [1]. While these changes are often well tolerated, patients with preexisting cardiovascular disease, particularly those with left ventricular hypertrophy (LVH) or outflow tract obstruction, may experience adverse cardiac remodeling and hemodynamic instability [1,2].

This case report describes a patient with severe LVH who developed recurrent hypotension and cardiac dysfunction following mature brachiocephalic AVF reopening. Echocardiographic findings revealed significant left ventricular outflow tract (LVOT) obstruction and mitral regurgitation, necessitating AVF closure. This case underscores the importance of individualized cardiovascular assessment in patients undergoing AVF procedures, particularly those with predisposing structural heart disease such as LVH.

## Case presentation

Our case involves a 56-year-old female patient with a history of hypertension, managed with amlodipine 5 mg and bisoprolol 5 mg, as well as end-stage renal disease, for which she underwent kidney transplantation 25 years ago. The donor was her non-human leukocyte antigen-identical sister, and the transplantation followed one year of dialysis. She has since been maintained on cyclosporine (Neoral) and prednisone.

She presented with a three-day history of dyspnea and decreased oral intake, complicated by acute kidney injury. Laboratory investigations revealed a markedly elevated creatinine level of 8.31 mg/dL, compared to her baseline of 4 mg/dL from the previous year, along with an increased blood urea nitrogen level, elevated liver enzymes, normal electrolyte levels, and elevated C-reactive protein. Hepatic viral screening, including hepatitis B virus, hepatitis C virus, hepatitis A virus, cytomegalovirus, and Epstein-Barr virus, as well as tests for COVID-19 and influenza A and B, was negative (Table 1). A chest radiograph showed an enlarged cardiac silhouette without evidence of pulmonary edema or infiltrates. Upon admission, the patient was hypotensive, with an oxygen saturation of 95% on room air. She was tachycardic (HR = 100 bpm) and tachypneic, and there was no evidence of pitting lower limb edema. Pulmonary auscultation revealed good bilateral air entry, while cardiac auscultation detected an S4 heart sound murmur at the apex.

**Table 1:** Laboratory investigation of our patient during hospitalization CRP: C-reactive protein, SGOT: serum glutamic-oxaloacetic transaminase, SGPT: serum glutamate-pyruvate transaminase, GGT: gamma-glutamyl transferase, Alk-Ph: alkaline phosphatase

Parameter	Patient value	Reference range
WBC	5710	4,500-11000/mm3
Hemoglobin	11.4 g/dl	13.5-17.5 g/dl
Platelets	71,600	150-400x109/L
Creatinine	8.31 mg/dl	<1 mg/dl
Sodium	137 mEq/L	135-145 mEq/L
Potassium	3.6 mEq/L	3.5-5.2 mEq/L
Chloride	95 mEq/L	95-105 mEq/L
CRP	108.3 mg/L	<5 mg/L
SGOT	158 IU/L	<50 IU/L
Alk-Ph	193 IU/L	<150 IU/L
GGT	154 IU/L	<65 IU/L
SGPT	160 IU/L	<35 IU/L

She was admitted to the critical care unit, where bisoprolol was discontinued. She received fluid boluses and was started on ceftriaxone for a urinary tract infection confirmed by urinary analysis and culture and norepinephrine for blood pressure support. Within 24 hours, she became anuric despite fluid resuscitation and developed hyperkalemia, requiring the placement of an intermittent dialysis catheter and initiation of dialysis. After three sessions of acute dialysis over six days, without signs of renal recovery, she stabilized and was weaned off vasopressors. Inflammatory markers had decreased, and she had no episodes of diarrhea or vomiting. Given her persistent need for dialysis, the decision was made to transition to continuous dialysis. A mature brachiocephalic AVF, which had been closed one year after her kidney transplantation for cosmetic reasons, was scheduled for reopening.

The AVF performed one week after stabilization was soft, easily compressible, and exhibited a normal thrill. During the augmentation test, a pulsation was observed in the vein. Six hours after the AVF reopening, the patient developed dizziness, dyspnea, hypotension, tachycardia, and a tonic-clonic seizure, requiring vasopressor support in the intensive care unit. These symptoms recurred during subsequent dialysis sessions, necessitating fluid boluses. As a result, cardiology was consulted for further evaluation. A prominent systolic thrill and a grade IV/IV murmur, easily audible at the apex and radiating to the axilla, were noted on physical examination.

**Figure 1:**
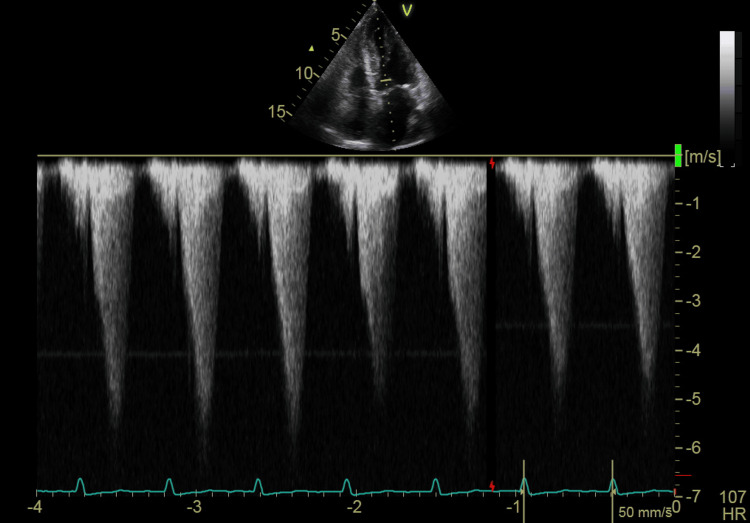
Continuous wave TTE at the LVOT obstruction and mitral valve showing a gradient of 103 mmHg at rest TTE: transthoracic echocardiography, LVOT: left ventricular outflow tract

**Figure 2:**
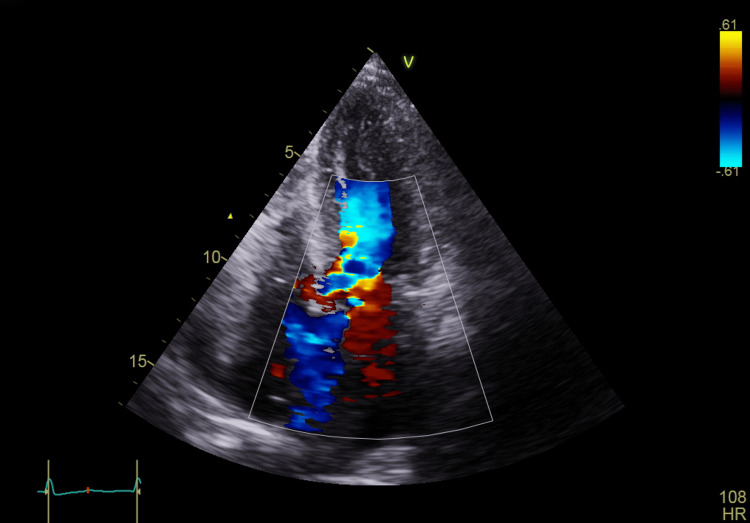
Apical four-chamber view showing LVOT obstruction with high-velocity turbulent flow LVOT: left ventricular outflow tract

A transthoracic echocardiogram (TTE) revealed a mildly dilated left atrium and severe concentric LVH (2.3 cm) with good contractility of all segments. The left ventricular filling pressure was elevated, and the left ventricular ejection fraction was 60%. The right atrium and right ventricle were normal in size, with normal right ventricular systolic function. The mitral valve showed grade 3 regurgitation, with SAM and LVOT obstruction, measuring a gradient of 103.42 mmHg. The aortic valve was calcified but showed no stenosis. The pulmonary valve appeared normal, while the tricuspid valve exhibited mild regurgitation. No pericardial effusion was noted (Figures 1-2, Video 1).

**Video 1:** LVOT obstruction observed on four-chamber TTE view LVOT: left ventricular outflow tract, TTE: transthoracic echocardiogram

**Figure 3:**
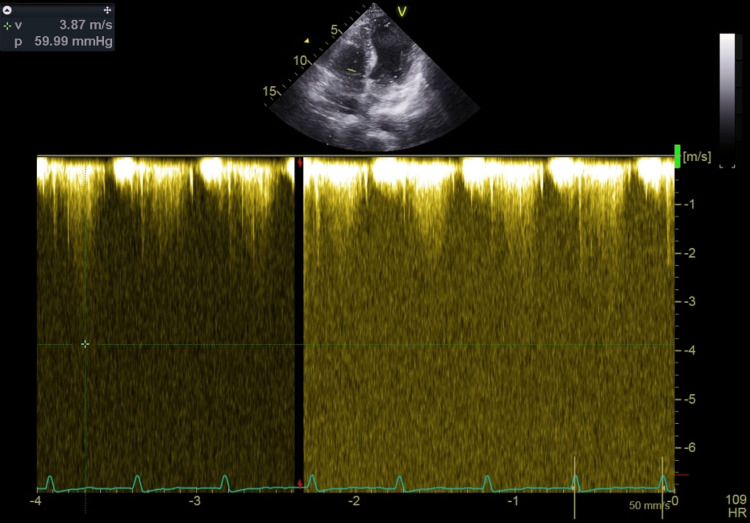
Continuous wave TTE at the LVOT obstruction and mitral valve showing a gradient of 59 mmHg after initiation of bisoprolol TTE: transthoracic echocardiography, LVOT: left ventricular outflow tract

The patient was started on the maximum dose of bisoprolol (10 mg). Still, symptoms persisted without improvement, and repeat echocardiography while on beta-blocker therapy revealed a reduction in LVOT gradient to 59 mmHg (Figure 3).

**Figure 4:**
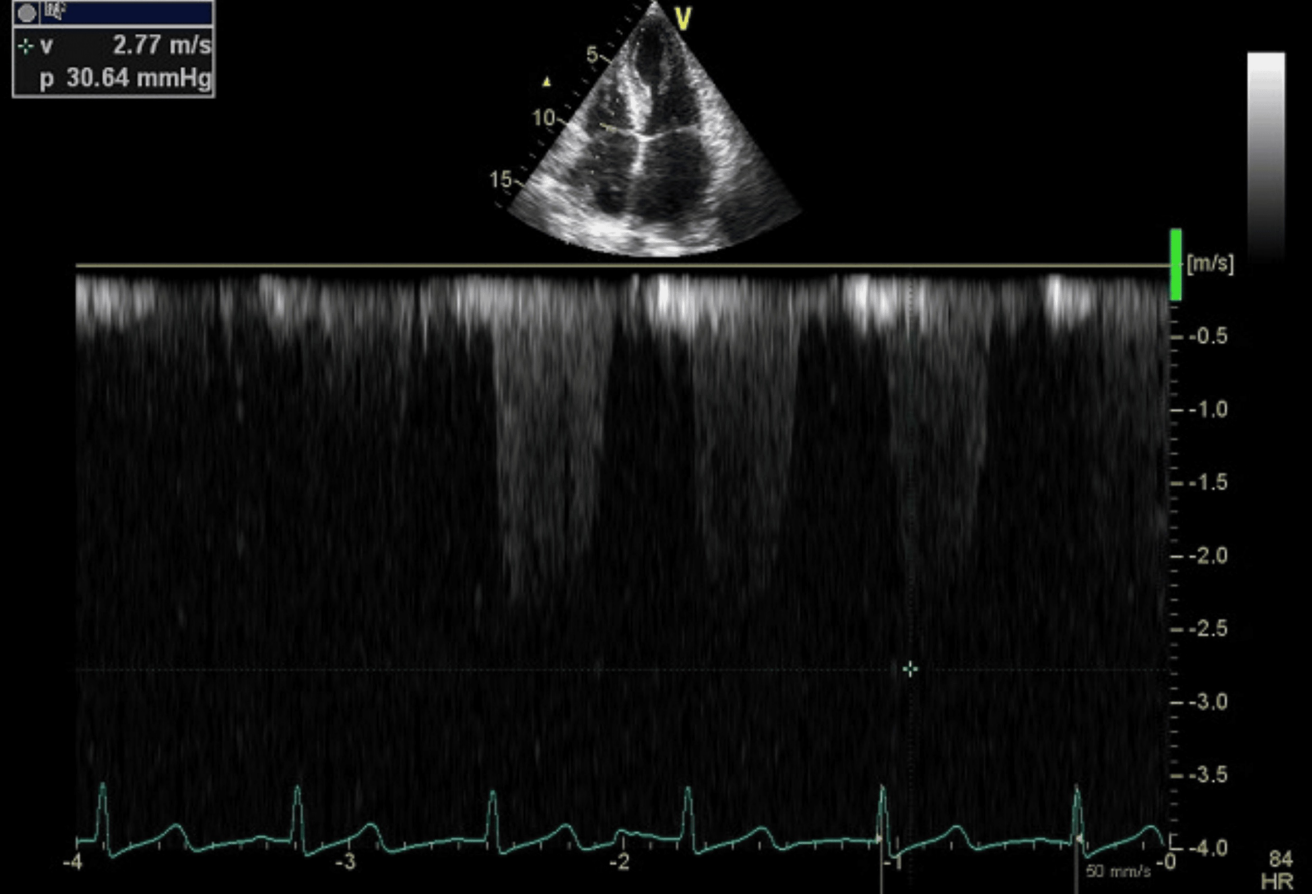
Continuous wave TTE at the LVH and mitral valve showing gradient of 30 mmHg after AVF closure TTE: transthoracic echocardiography, AVF: arteriovenous fistula, LVH: left ventricular hypertrophy

After multiple recurrent episodes, the decision was made to close the AVF. Following the procedure, the previously noted murmur resolved. TTE revealed an improvement in mitral regurgitation from grade III to grade I, along with the resolution of LVOT obstruction and a pressure gradient of 30 mmHg (Figure 4, Video 2).

**Video 2:** Four-chamber TTE view revealing resolution of SAM and obstruction following AVF closure TTE: transthoracic echocardiography, SAM: systolic anterior motion, AVF: arteriovenous fistula

## Discussion

AVFs have superior longevity, lower infection and mortality rates, and are associated with lower costs. Hence, they have become the vascular access for patients needing dialysis. Indeed, the prevalence of AVFs in the United States increased from 32% of all dialysis access in 2003 to 61% in 2012. Despite their association with lower mortality, AVFs significantly affect cardiac functions, which are predominantly related to the increase in preload and cardiac output [1].

Cardiac disease is the leading cause of mortality among patients undergoing chronic renal replacement therapy [2]. Compared to the general population, dialysis patients experience a significantly higher incidence of cardiovascular-related mortality and complications. This increased cardiac mortality is partially attributed to the high prevalence of preexisting cardiac disease before the initiation of dialysis. Additionally, the elevated burden of cardiovascular risk factors in patients with progressive kidney disease contributes to this increased mortality. Notably, among these patients, the prevalence of congestive heart failure (HF) is approximately 55%, arrhythmias occur in 46%, and other cardiovascular conditions affect 74%. Within this category, LVH is particularly common, with a prevalence of approximately 75% [2,3].

AVFs are considered the gold standard for vascular access in chronic hemodialysis due to their lower risk of long-term complications than AVGs and permanent cuffed catheters [4]. Despite their association with reduced mortality, AVFs can significantly impact cardiac function by increasing preload and cardiac output. In patients with end-stage kidney disease (ESKD), who already have a high burden of cardiovascular risk factors, this additional volume overload may contribute to adverse cardiac remodeling and the development of HF. A study by Reddy et al. involving 137 patients with ESKD undergoing AVF creation found that 53% had preexisting HF. In contrast, 43% of the remaining patients developed new-onset HF within a median of 2.6 years of hemodialysis initiation. Furthermore, patients who developed HF exhibited marked right ventricular dilatation and dysfunction, along with a 3.9-fold increased risk of mortality [5].

Creating an AVF results in the diversion of blood from the high-resistance arterial system into the low-resistance venous system, leading to an increase in venous return and cardiac output. Additionally, AVF formation reduces arterial impedance, thereby lowering left ventricular afterload. This reduction in arterial impedance may also decrease the effective circulating volume within the systemic circulation, triggering arterial baroreceptors and subsequently activating the sympathetic nervous system. This activation leads to increased cardiac contractility and further elevation in cardiac output [6].

One of the most significant complications associated with AVF creation, as reported in the literature, is the development of high-output HF due to excessive volume overload. Shunting arterial blood from the left to the right circulation increases preload and cardiac output, potentially causing cardiac hypertrophy and, over time, HF. Patients may exhibit signs such as tachycardia, elevated pulse pressure, hyperkinetic precordium, and jugular venous distension. Large AVFs, often in the upper arm, typically have blood flows exceeding 2000 mL/min. Echocardiography may show varying ejection fractions, while right-heart catheterization indicates elevated cardiac output with low to normal systemic vascular resistance [7]. This hemodynamic burden not only increases the risk of HF but also contributes to the development and progression of pulmonary hypertension, further exacerbating cardiovascular morbidity. Additionally, the chronic hemodynamic stress imposed by AVF can promote LVH in the long term, a well-recognized predictor of adverse cardiovascular outcomes in patients with ESKD [6,8].

A study by Lee et al. on mice found that AVF induced adaptive cardiac hypertrophy without causing functional decline or fibrosis. The research also revealed transcriptional changes linked to electrical remodeling and the upregulation of proangiogenic and prosurvival factors, indicating that AVF may have a cardioprotective yet potentially arrhythmogenic effect [9].

In this case, our patient, who had an LVH of 2.3 cm, developed recurrent episodes of hypotension following the reopening of a mature AVF. A cardiac examination revealed a prominent systolic thrill and an easily audible murmur at the apex, radiating to the axilla. Notably, this murmur was absent before the AVF was reopened and could still be heard with the stethoscope slightly off the chest. Transthoracic echocardiography demonstrated severe (grade 3) mitral regurgitation, SAM of the mitral valve, and LVOT obstruction, with a peak gradient of 103.42 mmHg, which is well above the threshold for severe obstruction (≥50 mmHg). A beta-blocker (bisoprolol 10 mg orally) was initiated; however, the patient continued to experience new episodes of hypotension, dizziness, and a decreased level of consciousness during each dialysis session. A repeat echocardiography while on beta-blocker therapy showed a reduction in LVOT gradient to 59 mmHg, which, while improved, remained within the pathological range (>30 mmHg), indicating persistent obstruction that likely contributed to ongoing symptoms and hemodynamic instability.

This decline in systemic vascular resistance likely resulted in a reduced effective circulating volume, stimulating arterial baroreceptors and initiating a compensatory response from the sympathetic nervous system. The observed hemodynamic changes were primarily driven by decreased arterial impedance following AVF reopening, leading to a hyperdynamic circulatory state. The sudden diversion of arterial blood flow into the venous system further reduced systemic vascular resistance, triggering a compensatory increase in myocardial contractility, which, in turn, exacerbated LVOT obstruction and contributed to hemodynamic instability. Additionally, dialysis-related factors, including rapid volume shifts, autonomic dysfunction, and uremia-related myocardial dysfunction, may have further contributed to episodes of hypotension and hemodynamic deterioration. Given the patient’s persistent instability despite medical management, a multidisciplinary discussion between the nephrology and cardiology teams led to the decision to surgically close the AVF.

Following the procedure, the previously noted murmur resolved. TTE revealed a marked improvement in mitral regurgitation from grade III to grade I, along with the resolution of LVOT obstruction and a pressure gradient reduction to 30 mmHg. Despite these improvements, the patient remained anuric. Given the ongoing risk of hemodynamic instability, the transition from intermittent to continuous dialysis was made to optimize volume control and minimize abrupt intravascular shifts. The patient’s next dialysis session was successfully performed using an intermittent catheter without complications. As part of the long-term management plan, a permanent dialysis catheter was placed while preparing the patient for peritoneal dialysis. This alternative strategy could help mitigate the cardiovascular burden associated with AVF-related hemodynamic changes.

While AVF closure was ultimately necessary, alternative strategies such as AVF banding, staged flow reduction, or endovascular interventions (e.g., coil embolization or balloon-assisted banding) could have been considered in select cases to reduce AVF flow without full closure. These approaches may be beneficial in cases where preserving some degree of AVF function is preferable while mitigating hemodynamic complications.

## Conclusions

AVFs are essential for long-term hemodialysis access, but their impact on cardiac function, particularly in patients with preexisting LVH, requires careful consideration. This case highlights how AVF reopening, unlike AVF creation, can precipitate acute hemodynamic instability due to the sudden reintroduction of high-flow dynamics, leading to LVOT obstruction and mitral regurgitation. Unlike a newly created AVF, a previously closed AVF lacks the gradual cardiovascular adaptation, resulting in an immediate and pronounced hemodynamic burden. In this patient, SAM-related mitral regurgitation further exacerbated the obstruction, and despite beta-blocker therapy, symptoms persisted, necessitating surgical AVF closure. Following the procedure, echocardiographic findings improved, and the patient could resume dialysis without complications.

This case underscores the importance of individualized cardiovascular assessment before AVF creation or reopening, particularly in patients with underlying cardiac pathology. A multidisciplinary approach involving nephrologists and cardiologists is essential to optimize patient outcomes and minimize the risk of hemodynamic complications. Key pre-procedural assessments, such as echocardiography, Doppler ultrasound, and dynamic LVOT evaluation, should be considered to identify at-risk patients. By incorporating these evaluations into clinical practice, AVF-related cardiovascular complications can be better anticipated and managed, ensuring both optimal dialysis access and cardiac stability.
